# Membrane Traffic in *Aspergillus oryzae* and Related Filamentous Fungi

**DOI:** 10.3390/jof7070534

**Published:** 2021-07-01

**Authors:** Yujiro Higuchi

**Affiliations:** Department of Bioscience and Biotechnology, Faculty of Agriculture, Kyushu University, 744 Motooka, Fukuoka 819-0395, Japan; y.higuchi@agr.kyushu-u.ac.jp; Tel.: +81-92-802-4734

**Keywords:** *Aspergillus oryzae*, endocytic pathway, filamentous fungi, *Koji* mold, membrane traffic, secretory pathway

## Abstract

The industrially important filamentous fungus *Aspergillus oryzae*, known as the yellow *Koji* mold and also designated the Japanese National fungus, has been investigated for understanding the intracellular membrane trafficking machinery due to the great ability of valuable enzyme production. The underlying molecular mechanisms of the secretory pathway delineate the main secretion route from the hyphal tip via the vesicle cluster Spitzenkörper, but also there is a growing body of evidence that septum-directed and unconventional secretion occurs in *A. oryzae* hyphal cells. Moreover, not only the secretory pathway but also the endocytic pathway is crucial for protein secretion, especially having a role in apical endocytic recycling. As a hallmark of multicellular filamentous fungal cells, endocytic organelles early endosome and vacuole are quite dynamic: the former exhibits constant long-range motility through the hyphal cells and the latter displays pleiomorphic structures in each hyphal region. These characteristics are thought to have physiological roles, such as supporting protein secretion and transporting nutrients. This review summarizes molecular and physiological mechanisms of membrane traffic, i.e., secretory and endocytic pathways, in *A. oryzae* and related filamentous fungi and describes the further potential for industrial applications.

## 1. Introduction

The filamentous fungus *Aspergillus oryzae*, known as the yellow *Koji* mold, is able to safely produce large amounts of valuable enzymes and metabolites and has been historically used in fermentation and brewing industries, typically in Japan [1,2,3]. Therefore, together with another yellow *Koji* mold *Aspergillus sojae*, the black *Koji* mold *Aspergillus luchuensis* and the white *Koji* mold *Aspergillus luchuensis* mut. *kawachii*, which are employed in soy sauce, awamori and shochu brewing, respectively, *A. oryzae* has been authorized as one of “The National fungi” of Japan [4]. However, the reason why *A. oryzae* cells can abundantly produce such valuable materials was not well understood with respect to molecular mechanisms of intracellular membrane traffic [5]. Since the completion of *A. oryzae* genome analysis in 2005, reverse genetic approaches have been widely applied to understand molecular mechanisms of both the secretory and endocytic pathways in the fungus [1,6]. Especially, fluorescent protein-based cellular biological analysis has greatly advanced the understanding of molecular machinery, especially about the predominant apical secretion [1,5]. Based on these findings, *A. oryzae* has been bred as a cell factory to produce valuable heterologous proteins and metabolites [7]. In addition, live-cell imaging revealed dynamics of endocytic organelles in *A. oryzae* hyphal cells [8]. This review summarizes molecular mechanisms of intracellular membrane traffic, mainly focusing on the secretory and endocytic pathways, in *A. oryzae* and its related filamentous fungi.

## 2. Secretory Pathway

### 2.1. Historical Studies of Secretory Proteins in A. oryzae

A hallmark of secretory proteins produced by *A. oryzae* is α-amylase, encoded by three almost identical genes *amyA/B/C*, with respect to the production quantity [9]. Historically, α-amylase, also known as Taka-amylase A from Takadiastase, was isolated and crystallized from *A. oryzae* cultures in the 1950s [10,11]. The enzymatic activity of α-amylase was found in both the culture medium and mycelium, suggesting that α-amylase is not only secreted to the medium but also localized to the cell surface [12]. Indeed, localization analysis by an indirect fluorescent-antibody technique using the antiserum against α-amylase demonstrated that α-amylase is located on the cell surface [13]. About a half-century later, recent analyses revealed that a cell wall component α-1,3-glucan is a potential inhibiting factor for α-amylase adsorption onto cell walls in the *A. oryzae* submerged culture [14]. In addition, pulse-chase experiments with L-[^35^S] methionine demonstrated a kinetic model of intracellular and extracellular α-amylase and also suggested that there exists fast or slow secreted α-amylase [15]. To investigate molecular mechanisms of an α-amylase secretion at the cellular biological level, AmyB has been generally selected because of its highest expression among *amyA/B/C* genes [16,17].

*A. oryzae* also secretes other carbohydrate hydrolases than α-amylase, including glucoamylase and α-glucosidase [2,9]. In addition, ribonuclease T1 (RNase T1), encoded by *rntA*, is secreted with guanosine-specific ribonuclease activity on single-strand RNA [18]. There are two secretory phospholipases (sPLAs) characterized in *A. oryzae*: PLA1-1 and sPlaA are phospholipase A_1_ and A_2_ that catalyze the sn-1 or sn-2 linkage of phospholipids, respectively [19,20]. There are 135 secretory protease genes predicted by the presence of signal peptide in the *A. oryzae* genome, among which *pepA* is a well-analyzed gene encoding acid protease [6,21]. The disruption of *pepA* enhanced the secretory production of heterologous proteins by avoiding degradation of the secreted proteins [22].

Solid-state culture (SSC) is a common industrial method to cultivate *A. oryzae* cells with cereal crops, such as rice, soybean and wheat [23]. Generally in *A. oryzae*, secretory proteins are more produced in SSC than in submerged culture [24]. In addition, there are certain proteins that are secreted specifically in SSC, but not in submerged culture; for example, a glucoamylase-encoding *glaB* is expressed and its protein is secreted only in SSC [25,26]. In contrast, another glucoamylase-encoding *glaA* is expressed in both SSC and submerged culture, but GlaA protein is secreted only in submerged culture [27,28]. These suggest that secretion of GlaB and GlaA is regulated at the transcriptional and posttranscriptional levels, respectively [24]. Moreover, as examples of industrial SSC, proteomic analyses on soy sauce fermentation using soybeans and wheat as the culture substrates identified extracellular proteases and amylolytic enzymes responsible for the generation of soy sauce flavors [29,30]. Furthermore, intriguingly, mixed cultures of *A. oryzae* together with another industrially important filamentous fungus *Aspergillus niger* grown in wheat bran produce a broader range of plant cell wall degrading enzymes compared with respective monocultures [31]. Due to stable interaction between *A. oryzae* and *A. niger* cells, this co-cultivation would have the potential for engineering enzyme cocktails.

### 2.2. Molecular Machinery of Secretory Pathway

#### 2.2.1. *N*-Glycosylation

Conventional secretory proteins harboring a signal peptide at the *N*-terminus are initially targeted to the endoplasmic reticulum (ER). From ER, these proteins are transported via Golgi to the plasma membrane by vesicular trafficking and lastly secreted to outside of cells. Through ER and Golgi, most of the secretory proteins are modified with *N*- and/or *O*-glycan chains, which have functions such as protein stability and localization [32,33]. Although the molecular mechanisms of both *N*- and *O*-glycans have been relatively well investigated in filamentous fungi, especially *N*-glycosylation mechanisms related to secretory proteins have been analyzed in *A. oryzae*. Systematic researches based on the genome information in filamentous fungi revealed the highly conserved machinery of *N*-glycosylation [33]. In the ER lumen, Glc_3_Man_9_GlcNAc_2_ (Glc, glucose; Man, mannose; GlcNAc, *N*-acetylglucosamine) are attached to Asn residue of glycoproteins, and thereafter glucosidases I and II remove Glc moieties [34]. These steps are known as the calnexin/calreticulin cycle for the quality control system of glycoproteins before transporting them to Golgi [35]. In addition, Man moieties are also cleaved off by two 1,2-α-mannosidases ManE and FmanIB at ER and Golgi, respectively [36,37,38]. As a secretory form of the *N*-glycan chain, Man_5_GlcNAc_2_ to Man_7_GlcNAc_2_, mainly Man_6_GlcNAc_2_, are attached on *A. oryzae* α-amylase [39]. The deletion of *Aooch1*, which putatively encodes a Golgi-localized α-1,6-mannosyltransferase, resulted in the reduced portion of higher-Man *N*-glycan onto secretory-produced human antibody adalimumab [40]. Moreover, by using an *A. oryzae* strain named AoGlycoDelete, in which endo-β-*N*-acetylglucosaminidase (ENGase) is expressed at the Golgi membrane to hydrolyze the linkage between the two core GlcNAc moieties of *N*-glycan, *N*-GlcNAc-proteins were produced extracellularly [41]. Unexpectedly, secreted *N*-GlcNAc-α-amylase exhibited normal enzymatic activity and thermal stability, suggesting that *N*-glycan of α-amylase does not affect the function of α-amylase. For the secreted *N*-GlcNAc-proteins, the remaining single GlcNAc moiety onto the glycoprotein might be important to maintain the protein structure and function.

#### 2.2.2. ER, Golgi and Spitzenkörper

**Figure 1:**
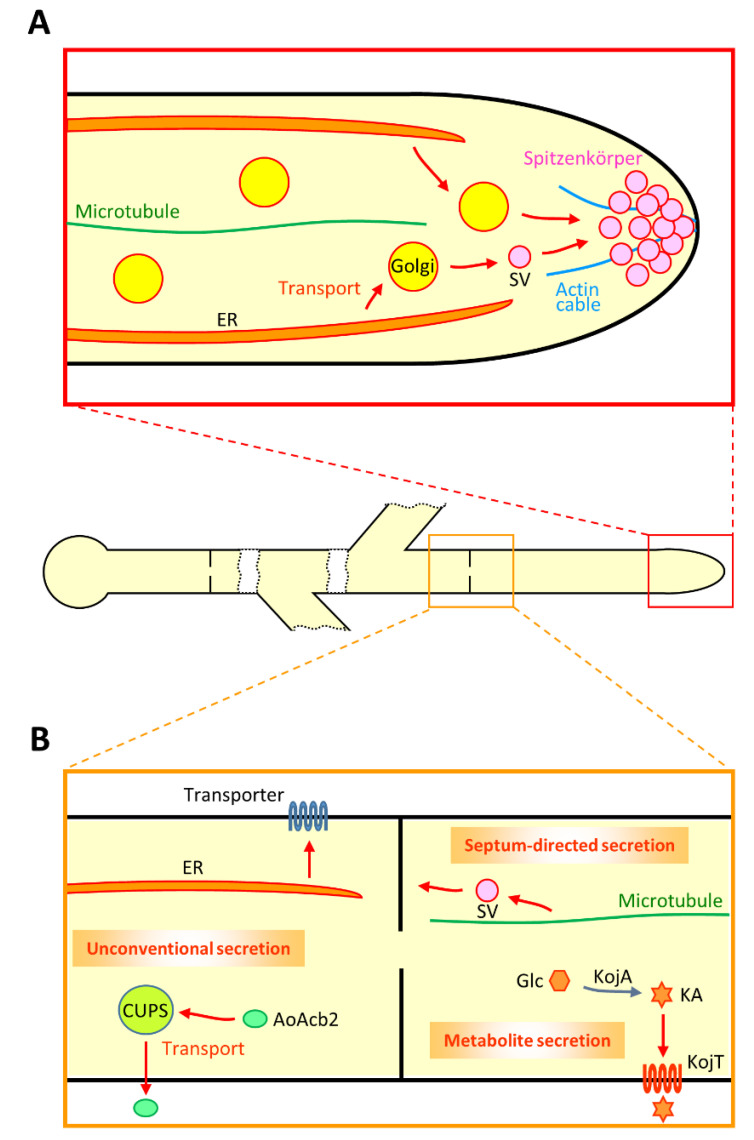
Secretory pathway in *A. oryzae*. (**A**) Secretion mechanisms at the hyphal tip region are depicted. Secretory proteins are transported via vesicles from ER through Golgi to the apical vesicle cluster Spitzenkörper. Apical secretion is thought to be dependent mainly on actin cable and supportively on the microtubule. SV, secretory vesicle. (**B**) Secretion mechanisms to the septum and plasma membrane around the hyphal middle region are shown. Septum-directed secretion is dependent on the microtubule but independent of the actin cytoskeleton, but further detailed molecular mechanisms are unknown. The cytoplasmic acyl-CoA binding protein AoAcb2 is unconventionally secreted under carbon starvation conditions, in which a compartment for unconventional protein secretion (CUPS) might be involved. Plasma membrane transporters are potentially transported directly from ER, not via Golgi. As an example of secondary metabolite secretion, kojic acid (KA) is synthesized from glucose (Glc) by the biosynthetic enzyme KojA and secreted by the putative plasma membrane transporter KojT. Note that the localization and transport of plasma membrane transporters do not appear to be restricted around the hyphal middle region.

To dissect the secretory machinery in *A. oryzae*, enhanced green fluorescent protein (EGFP)-tagged α-amylase AmyB and RNase T1 RntA were investigated for their subcellular localization [42,43]. In vivo imaging has revealed that the bright fluorescence of AmyB-EGFP and RntA-EGFP exists at the apical vesicle cluster Spitzenkörper, suggesting that these proteins are mainly secreted from the hyphal tip (Figure 1A) [42,43]. Moreover, both AmyB-EGFP and RntA-EGFP are also observed at septa, suggesting that there is molecular machinery for septum-directed secretion (Figure 1B) [44]. Indeed, fluorescence recovery after photobleaching (FRAP) analysis demonstrated that there is a constant flow of AmyB-EGFP to septa. Furthermore, secretion of AmyB-EGFP and RntA-EGFP to the hyphal tip is dependent on actin and microtubule cytoskeletons; in contrast, that of AmyB-EGFP to the septum is dependent on microtubule but independent of actin, suggesting that there are different molecular mechanisms between secretion to the hyphal tip and that to the septum [44].

In other filamentous fungi, there is a possibility that lateral secretion through the entire plasma membrane, not restricted from the tip and septum, might occur [45]. In fact, for *A. oryzae* cells, a recent report demonstrated that transient physical plasma treatment induces depolarization of the plasma membrane and activation of calcium ion influx into cells, resulting in increased α-amylase secretion [46]. Besides extracellularly secreted proteins, cell-wall-forming enzymes that are transported to the plasma membrane via vesicular trafficking have been well investigated [47]. In a model fungus *Ustilago maydis*, chitin synthases and 1,3-β-glucan synthase are transported in the same vesicle to the plasma membrane, suggesting that cell wall is synthesized locally by these enzymes, although whether such a transport mechanism exists in *A. oryzae* needs to be examined [48].

To understand the intracellular dynamics of secretory proteins, the subcellular localization of ER is crucial because the organelle is the initial part of the secretory pathway. Secretory proteins need to be properly folded in the ER lumen, where a chaperon protein BipA supports their folding. The subcellular localization of ER visualized by BipA-EGFP exhibited mesh-like structures in *A. oryzae* hyphal cells with dynamic motility and crowded composition to the tip region [49]. This localization pattern of ER suggests efficient protein secretion mainly from the hyphal tip. To further reveal the vesicular trafficking pathway from ER, a site of ER membrane called transitional ER (tER) was visualized by using an EGFP-fused marker protein AoSec13. tER exhibited punctate localization with a higher gradient to the tip, similar to ER localization [43]. In addition, lectin-like receptor proteins AoVip36 and AoEmp47 localized to ER-Golgi have been characterized. The deletion of *Aovip36* or *Aoemp47* improved heterologous protein secretion, suggesting that AoVip36 and AoEmp47 retain secretory proteins in ER and Golgi [50]. Furthermore, a genome-scale analysis suggested that an *A. oryzae* ortholog of *Saccharomyces cerevisiae* Erd2p that functions in the retrieval of ER-resident proteins from Golgi is involved in essential secretion machinery [51].

In the intracellular vesicular trafficking, vesicles need to be properly transported to the target membrane, in which soluble *N*-ethylmaleimide-sensitive factor attachment protein receptors (SNAREs) have important roles [52]. One vesicule-SNARE (v-SNARE) and three target-SNAREs (t-SNAREs) make a complex to allow the membrane fusion of a vesicle and the target membrane. Based on the genome information of the model yeast *S. cerevisiae*, reverse genetic analyses identified 21 SNAREs that existed in *A. oryzae* [53]. Comprehensive localization analysis of EGFP-fused SNAREs revealed that most of the proteins predictably reside at each membrane compartment; for instance, a v-SNARE AoSnc1 exhibits motility and localizes to secretory vesicles from Golgi to the plasma membrane and mainly at Spitzenkörper. Moreover, some t-SNAREs localize to septa, which is consistent with the existence of septum-directed secretion [44].

#### 2.2.3. mRNA Localization

*A. oryzae* α-amylase is not only abundantly secreted, but also α-amylase genes are highly transcribed, and the regulatory mechanisms of α-amylase genes have been well investigated [9]. As a negative feedback mechanism, the expression of α-amylase genes is repressed in the presence of glucose. It is also known that maltose is an inducing factor for the gene expression of α-amylase as well as other starch-degrading enzymes. Although biochemical mRNA expression analysis has been widely conducted and the molecular mechanism for α-amylase secretion has been well investigated as described above, little is known about the subcellular location of transcription and translation of α-amylase mRNAs in *A. oryzae* multicellular and multinuclear hyphal cells. 

To reveal the subcellular localization of α-amylase mRNAs in *A. oryzae* cells, single-molecule fluorescence in situ hybridization (smFISH) was recently conducted [54]. smFISH is one of the methods for mRNA localization analysis by using multiple fluorescent probes, which enable visualization of a single mRNA molecule [55]. smFISH with an *amyB*-specific probe demonstrated that the expression of α-amylase mRNAs was induced in the presence of maltose as the sole carbon source, but not glucose. Moreover, induced expression of α-amylase mRNAs with maltose addition was observed throughout the hyphal cells, suggesting the presence of α-amylase secretion not only at apical and septum but also at basal regions [54]. In contrast, actin mRNAs visualized by using a probe of actin-encoding *actA* are preferentially localized to the hyphal tip. Since the nucleus localized closest to the apex is generally more than 10 µm away from the tip, *actA* mRNAs might be actively transported from the nucleus to the hyphal tip where actin proteins are also localized [54].

### 2.3. Unconventional Protein Secretion

Although most of the proteins with signal peptides are thought to be secreted from the hyphal tip in filamentous fungi, there exist certain proteins lacking signal peptides that undergo unconventional protein secretion (UPS) [56,57]. In the yeast *S. cerevisiae*, an acyl-CoA binding protein Acb1 was found as a UPS protein that is secreted via a compartment for UPS (CUPS) and its molecular mechanisms have been well investigated [58,59,60]. Although there are only a few reports of UPS proteins available in filamentous fungi, a chitinase Cts1 of *U. maydis* is known to be unconventionally secreted in a lock-type manner [61]. During cytokinesis, Cts1 is secreted from the fragmentation zone formed between mother and daughter cells. In addition, Cts1 was successfully employed as a UPS carrier protein for heterologous protein production without modification of glycosylation onto the secreted heterologous proteins [62,63]. This provides further evidence that Cts1 bypasses ER and Golgi where glycosylation occurs. 

In *A. oryzae*, an acyl-CoA binding protein AoAcb2, one of *S. cerevisiae* Acb1 orthologs, was characterized as a UPS protein (Figure 1B) [64]. AoAcb2 that lacks signal peptide was found to be secreted under carbon starved conditions, but not under nitrogen starved conditions. Moreover, the UPS of AoAcb2 is dependent on the presence of the plasma membrane t-SNARE AoSso1, suggesting that AoAcb2 is secreted via vesicular trafficking. Furthermore, unlike the UPS machinery of *S. cerevisiae* Acb1, an autophagy-related protein AoAtg1 is not required for that of AoAcb2. These UPS properties of AoAcb2 are similar to those of a peptidase PepN in *A. niger* [65]. Further investigations are needed to understand detailed molecular mechanisms underlying the UPS pathway in *A. oryzae*. 

A recent analysis in *A. nidulans* reported that a model purine transporter UapA is transported from ER to the plasma membrane via Golgi bypass [66,67]. Investigation of neosynthesized UapA revealed this UPS pathway that is dependent on COPII vesicles, actin polymerization, clathrin heavy chain and the plasma membrane t-SNARE SsoA. Importantly, this UPS pathway of UapA is also applied to translocation of AzgA and FurA, purine and allantoin transporters, respectively. Whether such a UPS pathway for plasma membrane transporters, including AoUapC and AoGap1 that are known to be transported to the septum, exists in *A. oryzae* needs to be elucidated [44] (Figure 1B). 

### 2.4. Secretion of Metabolites

Recently, not only proteins but also certain metabolites were found to be secreted via intracellular membrane trafficking in filamentous fungi [68,69]. For example, *A. oryzae* extracellularly produces kojic acid (KA) as a secondary metabolite, which is used as a skin-lightening agent in cosmetics (Figure 1B) [70]. Although the KA biosynthetic processes are less understood, so far four KA biosynthesis-related genes—namely *kojA*, *kojR, kojT and kpeA*—were identified [71,72]. KojR and KpeA are Zn(II)_2_Cys_6_ transcriptional activator and repressor, respectively, that are thought to regulate the expression of *kojA* and *kojT* genes, which putatively encode an enzyme and a transporter, respectively. Indeed, overexpression of *kojA*, *kojR* or *kojT* and deletion of *kpeA* induce increased production of KA; in contrast, deletion of *kojR* abolishes KA production [72,73,74,75]. The expression of *kojA*, *kojR* and *kojT* genes is regulated by the global transcriptional regulator LaeA [76]. It is also known that KA production is sensitive to the presence of nitrate in the culture medium [71,77].

Although *A. oryzae* does not produce citric acid extracellularly, the black *Koji* mold *A. luchuensis* and its albino mutant *A. kawachii* can secrete plenty of citric acid [78,79]. Especially in *A. kawachii*, molecular mechanisms of citric acid secretion have been well investigated [80]. CtpA and YhmA are transporters localized to the mitochondrial membrane that transport citric acid from mitochondria to the cytoplasm [81]. Citric acid in the cytoplasm is used as a substrate to produce acetyl-CoA that is required for the biosynthesis of varieties of metabolites, such as lipids, amino acids and secondary metabolites. CexA is a plasma membrane transporter responsible for the extracellular secretion of citric acid from the cytoplasm, and the transcription of *cexA* is regulated by LaeA [82]. Although the *A. oryzae* genome holds two orthologs of *A. kawachii cexA*, these expression levels are low, which is consistent with the fact that *A. oryzae* does not produce citric acid extracellularly. Intriguingly, however, overexpression of *A. kawachii cexA* in *A. oryzae* cells resulted in citric acid secretion, suggesting that CexA is a limiting factor for citric acid secretion in the *Koji* fungi [83].

## 3. Endocytic Pathway

### 3.1. Historical Studies of Endocytic Pathway in A. oryzae

#### 3.1.1. Existence of Endocytosis in *A. oryzae*

Endocytosis is one of the conserved cellular processes that occur at the plasma membrane of eukaryotes for the acquisition of extracellular nutrients, internalization of plasma membrane proteins and reconstruction of cell polarity [84]. Most of the filamentous fungal genomes harbor homolog genes of endocytic proteins that were already identified in other eukaryotic cells [85]. However, in filamentous fungi, molecular mechanisms and physiological roles of endocytosis were not examined well. Moreover, the occurrence of endocytosis was elusive mainly because of the lack of reliable endocytic indicators [85]. As one of the initial applications for the investigation of endocytosis in filamentous fungi, the lipophilic dye FM4-64 was employed, which was generally used as an endocytic marker in yeast *S. cerevisiae* [86,87]. In addition, in *A. oryzae*, an EGFP-fused plasma membrane purine transporter AoUapC was utilized as another endocytic marker [88]. It was suggested that in the presence of ammonium in the culture medium, AoUapC-EGFP localized at the plasma membrane was no longer needed and underwent endocytosis. By using AoUapC-EGFP, it was also demonstrated that endocytosis occurs in ATP, temperature and actin-dependent manners in *A. oryzae* hyphal cells.

#### 3.1.2. Endocytic Recycling at the Hyphal Tip Region

To investigate physiological roles of endocytosis in *A. oryzae*, the function of *Aoend4*, the *A. oryzae* ortholog of *S. cerevisiae END4/SLA2*, the protein of which is an adaptor that connects the plasma membrane and actin cytoskeleton, was analyzed [89]. Since it was suggested that *Aoend4* is likely an essential gene for hyphal growth, a conditional mutant of *Aoend4* regulated by the *thiA* promoter was generated. Indeed, the repression of *Aoend4* resulted in defects of severe growth and endocytosis, analyzed by using FM4-64 and AoUapC-EGFP. Moreover, endocytosis-defective hyphae exhibited aberrant apical polarity. Hyphal elongation needs constant secretion via vesicles to the tip, and thus the v-SNARE AoSnc1 tagged with EGFP is mainly observed at the apical vesicle cluster Spitzenkörper. However, in *Aoend4*-repressed hyphae, EGFP-AoSnc1 was mislocalized to the whole plasma membrane, likely due to the lack of endocytosis. In addition, FRAP analysis at the tip region demonstrated that the apical recycling of EGFP-AoSnc1 was defective in *Aoend4*-repressed hyphae. These results suggest that endocytosis is crucial for apical growth and recycling of certain components required for vesicular trafficking [89]. Furthermore, transmission electron microscopy revealed that cell wall components were accumulated at large invaginated plasma membrane structures in endocytosis-deficient hyphae, suggesting that cell wall synthases also undergo endocytic recycling.

**Figure 2:**
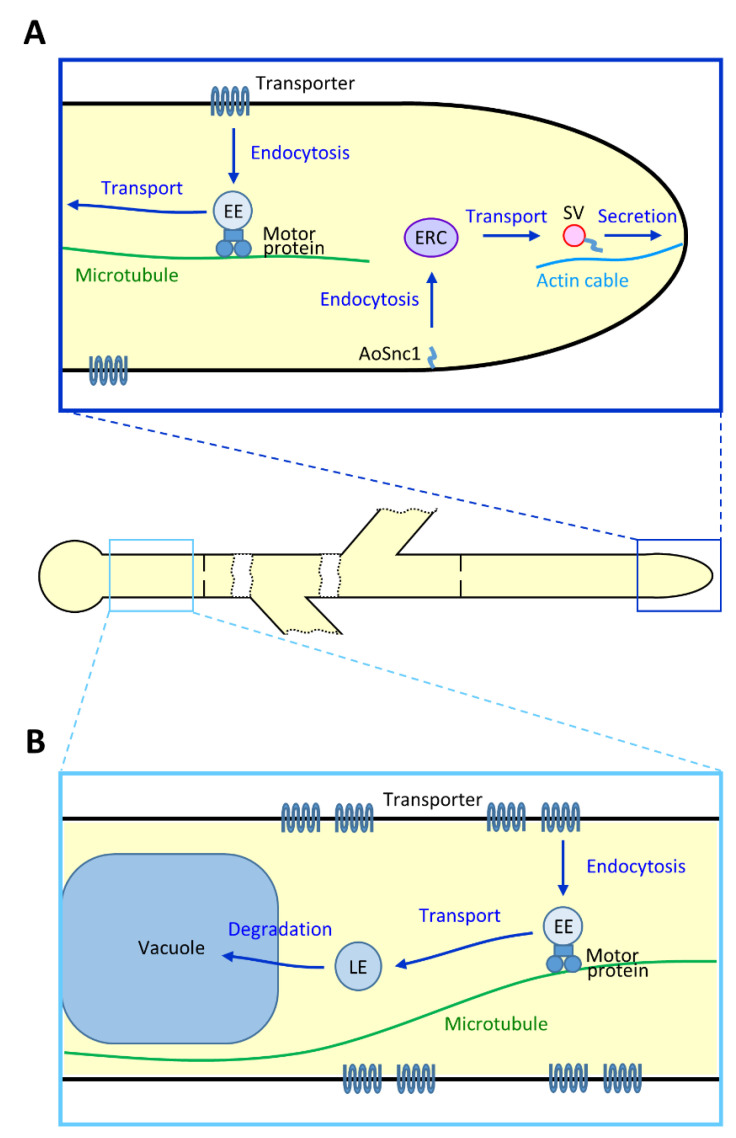
Endocytic pathway in *A. oryzae*. (**A**) Endocytic machinery at the apical region. The v-SNARE AoSnc1 is thought to be endocytosed from the plasma membrane and transported to the endocytic recycling compartment (ERC), although the existence of ERC has not been directly demonstrated in *A. oryzae*. Thereafter, AoSnc1 functions onto the secretory vesicle (SV). In contrast, plasma membrane transporters are endocytosed and transported through the early endosome (EE) for degradation. (**B**) Endocytosis at the basal region. Endocytosed plasma membrane transporters are transported via moving EE and relatively static late endosome (LE) and finally degraded in the vacuole.

Further localization analysis of AoEnd4 suggested that endocytosis actively occurs around the hyphal tip region, but is excluded slightly away from the apex [89,90]. Based on the investigations of the endocytosis-deficient mutant in *A. oryzae*, a model of endocytic recycling at the tip region was proposed (Figure 2A). In this model, proteins localized at the subapical plasma membrane, including v-SNARE AoSnc1, are endocytosed and transported to the endocytic recycling compartment (ERC), and thereafter are recycled via secretory vesicles to Spitzenkörper. *A. oryzae* can secrete large amounts of proteins, such as α-amylase, to the medium and this apical endocytic recycling mechanism may support such enormous secretion capacity. The endocytic recycling model has been widely accepted in filamentous fungi, and other recycling proteins have been identified [47,91,92,93]. Collectively, apical endocytic recycling is closely connected with continuous growth and secretion at the hyphal tip region.

#### 3.1.3. Other Molecular Mechanisms in Endocytosis

**Figure 3:**
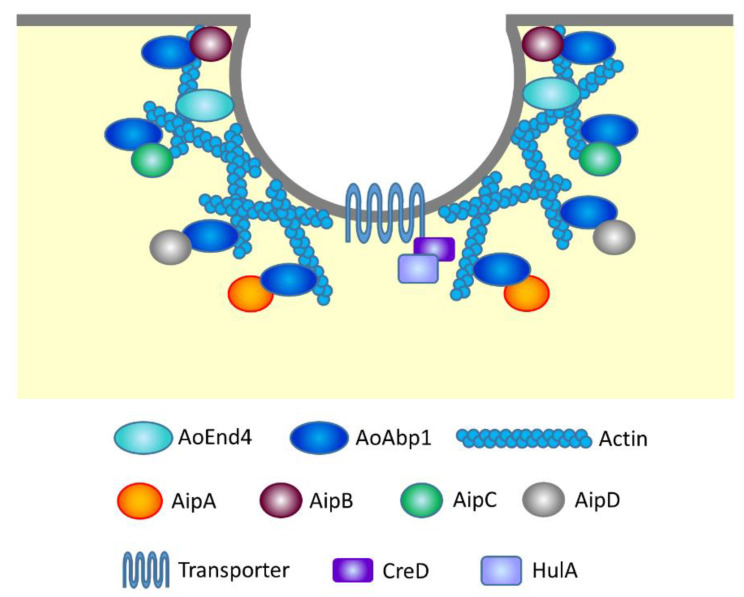
Schematic diagram of endocytic proteins involved in transporter endocytosis in *A. oryzae*.

It is well known that actin and its related proteins are involved in endocytosis by forming actin patches that generate force to pull the plasma membrane into the cell’s interior [94]. In this context, the localization of AoAbp1, the *A. oryzae* ortholog of *S. cerevisiae* actin-binding protein Abp1, was investigated [89]. Similarly to AoEnd4, AoAbp1 localizes at the subapical, collar of the hyphal tip. *S. cerevisiae* Abp1 possesses one SH3 domain that functions in protein interaction, whereas AoAbp1 harbors two SH3 domains. This difference raised the possibility that there exist discrete protein interactions with AoAbp1, which are not found in *S. cerevisiae* Abp1. Therefore, yeast two-hybrid screening using AoAbp1 as bait and *A. oryzae* total library proteins as prey was conducted, and four AoAbp1-interacting proteins AipA, B, C and D were identified [95]. All these Aip proteins exhibited typical endocytic collar localization as expected [95,96]. AipA is a putative AAA (ATPases associated with various cellular activities) ATPase and the overexpression of *aipA* induces a defect of FM4-64 endocytosis at the tip region, suggesting that AipA is a negative regulator of endocytosis [95]. AipB is predicted to encode class I myosin heavy chain, which orthologs are Myo3 and Myo5 in *S. cerevisiae*, and is essential for endocytosis and growth [96]. AipC is the ortholog of *S. cerevisiae* actin patch protein App1, and no AipD ortholog is found in yeast. Although AipC and AipD are dispensable for endocytosis of FM4-64, these proteins are required for endocytosis of the arginine permease AoCan1 that appears to localize at the membrane compartment of Can1 [96] (Figure 3). 

A maltose permease MalP localized at the plasma membrane has been analyzed as a protein that undergoes endocytosis in *A. oryzae* [97]. In the presence of maltose in the medium, MalP at the plasma membrane incorporates maltose to induce the expression of amylolytic enzyme genes. In contrast, in the presence of glucose, MalP is endocytosed and the expression of amylolytic enzyme genes and *malP* is repressed. The internalization of MalP is regulated by the ubiquitin ligase HulA, the ortholog of *S. cerevisiae* Rsp5 [97]. In addition, an arrestin-like protein CreD is thought to function as an adaptor of MalP and HulA and is involved in glucose-induced endocytosis of MalP [98]. Whether endocytosis of other plasma membrane transporters, such as AoUapC and AoCan1, is also dependent on HulA and/or CreD needs further investigations (Figure 3).

#### 3.1.4. Molecular Machinery Related to Endocytic Organelles

Endocytosed proteins are first transported to early endosome (EE), then to late endosome (LE) and finally to the vacuole for degradation (Figure 2B). To characterize these endocytic organelles, SNARE and small GTPase Rab proteins have been employed in *A. oryzae* [53,99]. In mammalian cells, EEs are maturated to LEs by conversion of Rab5 to Rab7, the molecular mechanisms of which are thought to be conserved in filamentous fungi [100,101]. In filamentous fungi, Rab5-positive EEs are highly motile and move through the cell; by contrast, Rab7-positive LEs are mostly static and in general observed adjacent to vacuoles [99,101,102]. EEs motility is a hallmark of endocytic organelles in filamentous fungi and its underlying molecular mechanisms are described in the following section.

The vacuole is the destination of the endocytic pathway. To properly transport certain proteins, including proteases, into vacuoles, there is the molecular machinery of vacuolar protein sorting (Vps). In *S. cerevisiae*, mutants of *VPS* genes were identified by the screening using carboxypeptidase Y (CPY) as a Vps cargo, in which CPY was not transported to vacuoles but missorted to the medium [103,104,105]. Accordingly in *A. oryzae*, *VPS* mutants were visually isolated by using CPY-EGFP [106]. Among these mutants, AoVps24 was characterized as a component of the endosomal sorting complex required for transport (ESCRT)-III [107]. Together with ESCRT-I and ESCRT-III components AoVps23 and AoSnf7, respectively, AoVps24 exhibited LE-like localization [108]. The deletion of *Aovps24* resulted in defects of proper vacuolar formation and mycelial growth, and the same phenotypes were observed in the deletion of another ESCRT-III gene *Aovps2*, suggesting that ESCRT-III components are required for vacuolar formation that is essential for mycelial growth [108].

### 3.2. Dynamics of Endocytic Organelles

#### 3.2.1. Early Endosome

In *A. oryzae* hyphal cells, EE dynamics were firstly visualized with AoUapC-EGFP after the induction of endocytosis by the addition of ammonium to the medium [88]. EEs exhibited long-range motility through the hyphal cell, and its motility was perturbed by a microtubule (MT)-depolymerizing reagent nocodazole. The detailed molecular machinery underlying the constant long-range EE motility has not yet been characterized in *A. oryzae*, but well understood especially in other model filamentous fungi *U. maydis* and *A. nidulans* [109,110,111]. EEs move bidirectionally along MTs by motor proteins kinesin-3 and dynein towards MT plus and minus ends, respectively [112,113,114,115,116]. There is an adaptor protein complex Fused Toes (FTS)/Hook/ FTS and Hook-interacting protein (FHIP) (FHF) between EE and motor proteins [117,118,119]. In the mutants of Hook, EEs become immotile but motor proteins still move [117].

EEs visualized with EGFP-tagged AoRab5, the Rab5 homolog in *A. oryzae*, exhibit constant motility through the cell, not restricted when endocytosis is induced [99]. This observation raised the possibility that EEs have other physiological roles than endocytic function. Indeed, investigations in *U. maydis* revealed that constant EE motility distributes translationally active polysomes and other organelles, such as ER, peroxisome and lipid droplet in hyphal cells [102,120]. Moreover, long-range EE motility has a role in signal transduction in the process of plant infection [121]. To elucidate further physiological roles of EE motility, the deletion mutant of *Aohok1*, the Hook ortholog in *A. oryzae*, was investigated [99]. In the *Aohok1* disruptant, levels of transcripts and secreted proteins of α-amylase are reduced, suggesting that EE motility has roles in gene expression and secretion for α-amylase in *A. oryzae*. Similarly, in the EE motility-defective *rabB^rab5^* mutant of *A. nidulans*, the secretion of a glycosyl hydrolase inulinase InuA was impaired [122]. Taken together, EE motility has various physiological functions in filamentous fungi and further investigations are required to elucidate whether EE motility is involved in signal transduction in *A. oryzae*. 

#### 3.2.2. Vacuole

The vacuole is an acidic organelle and has crucial physiological roles, such as storage of metabolites and regulation of cytoplasmic homeostasis [123]. Visualization of vacuoles in *A. oryzae* cells was initially conducted with CPY-EGFP that localizes in the vacuolar lumen and demonstrated pleiomorphic vacuolar structures [124]. However, since fluorescence of CPY-EGFP in vacuolar lumen varied depending on culture pH, EGFP-fused t-SNARE AoVam3 that localized to the vacuolar membrane was employed to stably visualize vacuoles [124,125]. Indeed, visualization of EGFP-AoVam3 revealed that vacuoles are highly dynamic, some of which exhibit not only spherical and cubic structures but also moving small punctate and tubular structures [125]. Especially such tubular vacuoles are thought to be involved in intra- and intercellular transport of nutrients [126].

Autophagy is a key physiological event that occurs in vacuoles under nutrient starvation conditions to maintain cellular homeostasis [127]. *A. oryzae* is one of the model filamentous fungi to study autophagy machinery. In fact, in multinuclear *A. oryzae* cells, the dynamic autophagic process of nuclei was firstly found among eukaryotes and this phenomenon was designated macronucleophagy, which is induced under carbon or nitrogen starvation conditions [128,129]. Moreover, autophagy might be involved in supporting nutrient transport from vacuoles of basal hyphal regions via tubular vacuoles to aerial hyphal cells where nutrients are not supplied from the cell exterior through the plasma membrane [130,131]. Molecular components of AoAtg proteins involved in *A oryzae* autophagy have been well summarized previously [5]. Recent analyses further identified AoAtg11 and AoAtg26: the former functions in the selective autophagy of peroxisomes and mitochondria, and the latter is involved in autophagic degradation of organelles in vacuoles [132,133]. Moreover, related to autophagy machinery, an acyl-CoA binding protein AoAcb1 exhibits long-range motility in the cytoplasm and its subcellular localization is regulated by autophagy proteins; however, the physiological importance of the regulation on AoAcb1 localization remains yet unknown [134].

## 4. Conclusions and Perspectives

In this review, historical studies of membrane traffic in the industrially important filamentous fungus *A. oryzae* and its related filamentous fungi are summarized. Due to the great ability of valuable enzyme secretion, the molecular mechanisms of the secretory pathway were investigated in *A. oryzae* [1]. Based on the findings, *A. oryzae* was bred as a cell factory to extracellularly produce heterologous proteins [7]. Since the removal of *N*-glycans of heterologous glycoproteins increased the enzymatic activity, such as chymosin produced by *A. niger* and cellobiohydrolase produced by *Trichoderma reesei* [135,136], AoGlycoDelete would be a useful strategy for secretory production of heterologous glycoproteins [41]. Although *A. oryzae* SSC has been widely utilized in fermentation and brewing industries, cellular and physiological studies on *A. oryzae* SSC have not yet been conducted well. Recently, it was revealed that transcriptional and translational heterogeneity in multicellular *A. oryzae* cells is related to protein secretion in stress responses [137]. Moreover, it was shown that under stress conditions, stress granules that consist of non-translating messenger ribonucleoproteins are formed around the hyphal tip region of *A. oryzae* cells [138], although the underlying molecular and physiological details of stress granule formation need further analyses. Since submerged culture and SSC exhibit different regulations of transcription and translation of certain enzymes, elucidation of such molecular mechanisms would be beneficial for further effective industrial use of *A. oryzae* cells. On the other hand, not only the secretory pathway but also the endocytic pathway was found to be important to support apical secretion [90]. In addition, EE dynamics also contribute to protein secretion and might be involved in protein synthesis [99]. Furthermore, certain SMs in filamentous fungi are synthesized in specific organelles [69], and thus indeed optimizing the subcellular localization of biosynthetic enzymes enhanced penicillin production [139]. Therefore, novel findings on membrane traffic related to SM biosynthesis would provide a new strategy for improving valuable SM production in *A. oryzae* [140]. Finally, because genome editing was successfully applied to *A. oryzae* [141,142], molecular manipulation based on discoveries in membrane traffic would be favorable, especially onto industrial strains for further valuable material production. 

## Data Availability

Data are available within the manuscript.
